# Location of centers of rotation and existence of terminal hinge axes during jaw movements – a preliminary in vivo clarification

**DOI:** 10.1007/s00784-025-06619-4

**Published:** 2025-10-25

**Authors:** Albert C. Mehl, Sarah C. Woodford, Dale L. Robinson, Jaafar Abduo, Peter V. S. Lee, David C. Ackland

**Affiliations:** 1 Division of Computerized Restorative Dentistry, Center of Dental Medicine, University of Zurich, Plattenstrasse 11, Zurich, 8032 Switzerland; 2 Department of Biomedical Engineering, University of Melbourne, Parkville, Victoria 3010 Australia; 3 Melbourne Dental School, University of Melbourne, Parkville, Victoria 3010 Australia; 4 Department of Mechanical and Mining Engineering, University of Queensland, St Lucia, Queensland 4072 Australia

**Keywords:** Temporomandibular joint, Mouth opening, Hinge axis, Center of rotation, Jaw kinematics

## Abstract

**Objectives:**

This study aimed to evaluate the behaviour of jaw opening movements in vivo, especially focusing on locating the center of rotation and extracting the translational and rotational components of mandibular movements respectively.

**Materials and methods:**

Jaw movements of healthy participants were recorded using an optoelectronic tracking device and kinematics registered to bony anatomy segmented from CT scans. An evaluation program was developed to support visualization and quantification of mandibular movements. Landmarks representing anatomic and arbitrary condylar positions were defined and their trajectories investigated. Center of rotation positions were calculated for the full opening paths, and rotation-translation diagrams were recorded to investigate condylar movements for different jaw opening angles.

**Results:**

Analyzing landmark trajectories in a virtual skull model explicitly demonstrated their dependency on the spatial 3D location relative to the condyle center. For the full mouth opening, the center of rotation averaged 32.4 (± 7.4) mm caudally and 0.2 (± 10.8) mm posteriorly to the condyle center. These values aligned well with a theoretical calculation assuming combined translational and rotational condyle movement. A combined condylar rotational and translational movement could also be demonstrated using the rotation-translation diagrams, indicating that no pure rotation around the condyle was observed for the initial mouth opening movement.

**Conclusion:**

This in vivo study indicates that the assumption of a pure condyle rotation during initial mouth opening is not supported by the data. As this assumption underpins standard mechanical articulator concepts, these concepts need critical reevaluation.

## Introduction

Functional aspects of jaw movements are important for many treatment and diagnostic purposes. At present, however, the measurement of functional parameters is still primarily carried out without considering patient specific anatomical structures such as condyle position, condyle shape and the surrounding limiting morphology. In particular, for most measuring devices, posterior so-called arbitrary axis points that are close to the anatomical condyles are used as reference points in a standard protocol [1]. From these arbitrary axis points, conclusions are drawn regarding the real movement of the anatomical condyle [1]. However, the choice of condylar reference points for the analysis of the jaw movements may influence the interpretation of the pathways and can result in diagnostic errors [2, 3]. An alternative reference is to use the terminal hinge axis (THA), an axis which assumes a pure rotation of the mandible during the initial part of the mouth opening. Despite the assumption of having an axis of pure rotation and the position is a priori not related to the condyle anatomy, the THA has been found to be located near the condyle centers [4].

The position of the THA or the arbitrary hinge axis has played a central role in the function of mechanical articulators. For that, mandibular movement is assumed to occur through a combination of rotations and translations of those axes [5]. Thus, programming a mechanical articulator requires identifying an axis of rotation (e.g. individual and arbitrary terminal hinge axis) and quantifying the angles and translational shifts of specified axis movements (sagittal inclination, Bennett angle, immediate side shift etc.) [6]. Mandibular movement can thus be prescribed using these aforementioned parameters.

New technologies have gained popularity for rapidly segmenting medical images into 3D anatomical models, which can subsequently be registered to electronic jaw movement data. This approach can support detailed kinematics of the jaw relative to structural anatomy [7–9]. Despite this capability, there remains debate as to the existence of a hinge axis, or the presence of a pure condylar rotation during initial opening movements [10]. Few studies have used dynamic 3D models for investigating this fundamental assumption in vivo [11–13]. These include studies of the fixed center of rotation (CoR), instantaneous centers of rotation (ICR), helical axes and the movement of anatomical condyle axes.

Another aspect requiring deeper insight into 3D jaw movements is the design of a prosthesis for total joint replacement (TJR) of the temporomandibular joint (TMJ). The movement of a TMJ reconstructed by means of a TMJ-TJR prosthesis has predominantly supported rotational movement [14]. Translational movement of a prosthetic TMJ is restricted due to a lack of lateral pterygoid muscle function. It is therefore sometimes recommended to choose a fixed CoR, and reduce the free translation of the neo-disc as much as possible [7]. In general, positioning the joint closer to the CoR could significantly reduce the required translation rail lengths, potentially making the design of TMJ prostheses more feasible while still accommodating all functional aspects [8]. A previous study found that the fixed CoR for a mouth opening in healthy adults is located far inferiorly and posteriorly to the condyle center [7]. Interestingly, these locations seem to roughly align with findings from a fundamental kinematic analysis, which assumed a linear correlation between translation and rotation of condyle movement [15]. This suggests that pure rotational movements during mouth opening are rare, though this has not been proven to date.

The aim of this in vivo study was to evaluate jaw movements in healthy individuals by combining optoelectronic motion data with subject specific anatomy derived from CT scans. A visualization tool was developed to reproduce the movements of the mandible and visually interpret the trajectories of arbitrary reference points around the condyle centers and the incisal points in a virtual skull model. Following this, the CoR during mouth opening movements was determined from the measurements and compared to a theoretically derived formula. Finally, rotation-translation diagrams were created to assess condylar movement for evidence of pure rotation.

## Material and method

### Participant recruitment and motion analysis experiments

Eight healthy participants with nearly full dentition and no signs or history of TMJ disorders or other functional restrictions were recruited. Participant recruitment and data acquisition was based on an investigation conducted at the University of Melbourne and is described in detail in Woodford et al. (2023) [8]. All participants had functional dentition at the time of testing, and were excluded if they had existing health conditions, experienced dental pain, were pregnant or breastfeeding, or had a history of orthodontic treatment. Ethical approval was obtained from the institutional Human Ethics Advisory Group (HREC 1853328.1), and all participants provided written informed consent. A cone-beam computed tomography (CBCT, Aquilion One, Canon Medical Systems, USA) scan with an isotropic voxel size of 0.4 mm was taken of each participant’s mandible, maxilla and both TMJs. Imaging was performed with the participant lying on their back with their mandible in the maximum occlusion position. The CBCT images were digitally segmented using threshold-based segmentation (Mimics, Materialise, Belgium) and 3D models of each participant's mandible and skull, including dental anatomy, were reconstructed using commercial software (3-Matic 13.0, Materialise, Belgium).

Pairs of lightweight subject-specific motion tracking plates that attach to each participant’s mandibular and maxillary dental arches were developed to rigidly support triads of retro-reflective markers for dynamic optical motion analysis of the mandible (Röhrle et al., 2009). Before use, the plates were mounted onto dental stone casts of each participant and digitised using a handheld laser scanner with an accuracy of 0.05 mm (Artec Spider, Artec 3D, Luxembourg). The wire clasps on each plate were positioned posteriorly to allow complete occlusion of the teeth and closure of the lips without obstruction. The dental clasps also provided stable mounting points for laser welded wire extending extraorally to support the retroreflective markers. A 3D stereolithography (stl) surface model was generated for each motion tracking plate using 3-matic software in order to register the retro-reflective marker positions to the occlusal anatomy.

Each participant was seated with custom motion tracking plates fitted to their maxillary and mandibular dental arches. Participants were instructed to perform in random order, five cycles of maximum mouth opening, maximum protrusion, and maximum lateral excursions towards the left and right side. During tasks, the trajectories of the six retroreflective markers were simultaneously recorded using a 4-camera video motion analysis system (Vero, Vicon, Oxfordmetrics, UK) sampling at a frequency of 100 Hz. All motion data were filtered using a lowpass filter with a cut-off frequency of 10 Hz. This technique captured jaw motion measurements with a precision of 0.13 mm (Woodford et al. 2023) [8].

### Kinematic data evaluation

**Fig. 1:**
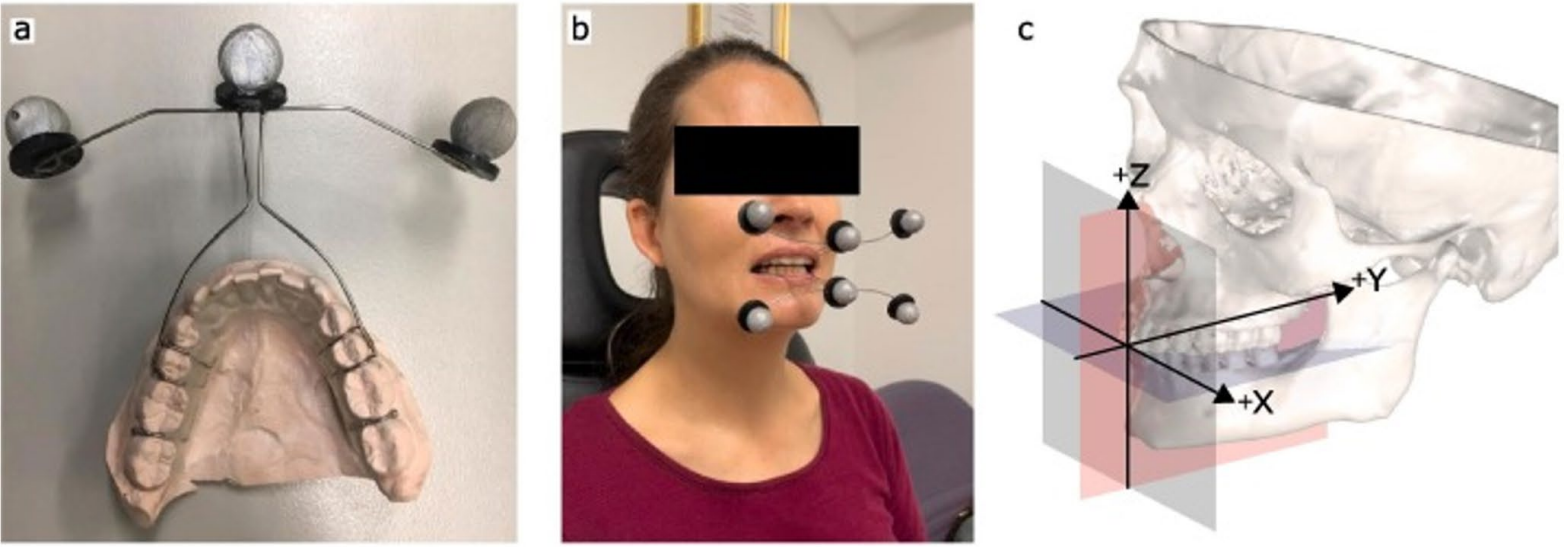
Design of the custom-made motion tracking plate fixed to its dental stone cast illustrating three retroreflective markers, clear lingual base plate, metal clasps around the second premolars and molars, and wire extending extraorally connecting the clasps to the retroreflective markers (**a**), participant fitted with maxillary and mandibular tracking plates (**b**), and anatomical coordinate system used to define mandibular motion, with the occlusal, sagittal, and frontal planes displayed in blue, red and grey, respectively (**c**). Positive X, Y and Z directions correspond to the lateral (left), posterior and superior directions, respectively

The jaw motion data were registered to the segmented skull and mandible by superimposing the laser scanned dental casts with the segmented teeth from the CBCT scan. In order to have a standardized anatomical coordinate system referenced to the skull, all mandibular motion data were then referenced to the occlusal plane, which was defined by the maxillary incisal edges and the cusps of the posterior maxillary molars, the sagittal plane defined perpendicular to the occlusal plane passing through the maxillary interincisal point and the midpoint between the left and right condylar center points (see below), and the frontal plane perpendicular to the occlusal and sagittal planes (Fig. 1c). The recorded jaw kinematics was transformed to this reference coordinate system, resulting in a sequence of transformation matrices describing the recorded mandible movements in relation to a fixed maxillary and skull.

All data processing and visualization was completed using Matlab (Matlab R2024a, Mathworks). The segmented skull and mandible models including the dentition and the condyles were imported as stl files. The sequence of transformation matrices describing the motion of the mandible with respect to the skull were also imported and categorized into the different jaw movements (opening, protrusion and left and right excursions). With a custom-developed visualization program, the different motions could be visualized at any speed and for any desired movement.

### Reference point trajectories

Using both an interactive tool and with specific calculations, test reference points were defined and the movement traces for each point was recorded and displayed in the occlusal plane (x-y-plane), the frontal plane (x-z-plane) and the right and left sagittal plane (y-z-plane). Trajectories of the following landmarks were analyzed during mandibular movement (i) Incisal point defined as the middle of the mesial incisal edges of the first lower incisors, and (ii) condylar center points, constructed as follows: The lateral and medial condylar poles were identified on each side of the mandible and the right and left mid-point were calculated, defining the right and left condyle point.

**Fig. 2:**
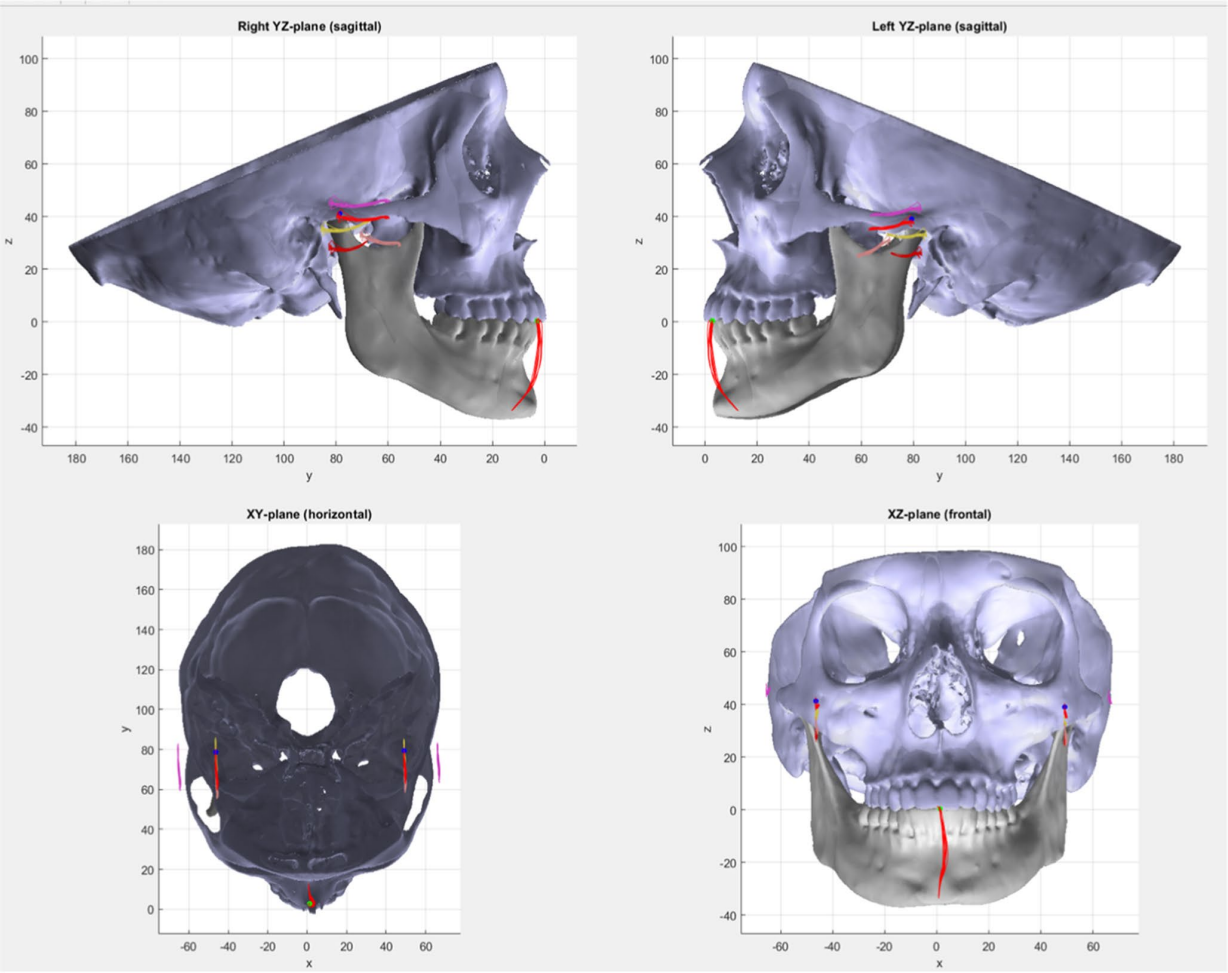
Projections of the virtual skull model to the respective planes: sagittal from right, sagittal from left (both yz-planes), horizontal (occlusal, xy-plane) and frontal (xz-plane). For the mouth opening movement, the trajectories of the condyle centers and incisal point (red lines) and the 4 reference points (coordinates see material and method section) are shown

To investigate the influence of condylar position errors on the movement traces and on parameters such as sagittal inclination, length of trace etc., trajectories of the following test points were recorded as displacements in relation to the condyle center point (in mm):

1. Caudal-Lateral: ∆x= -/+18; ∆y = + 3; ∆z = + 5; (∆x to simulate position on skin)
2. Cranial: ∆x = 0; ∆y= +3; ∆z= −12;
3. Dorsal: ∆x = 0; ∆y = + 6; ∆z= −5;
4. Ventral: ∆x = 0; ∆y= −9; ∆z= −7;

The displacement values were chosen to reflect the errors associated with determining the terminal hinge axis using arbitrary face bows [4, 16]. The trajectories of all points were visually analyzed and systematic differences in comparison to the trajectory of the condylar center points were noticed. For the visual evaluation, the trajectories were displayed together with the anatomical structures of the skull and the mandible in each projected plane and qualitatively analyzed (Fig. 2).

### Center of rotation calculations

**Fig. 3:**
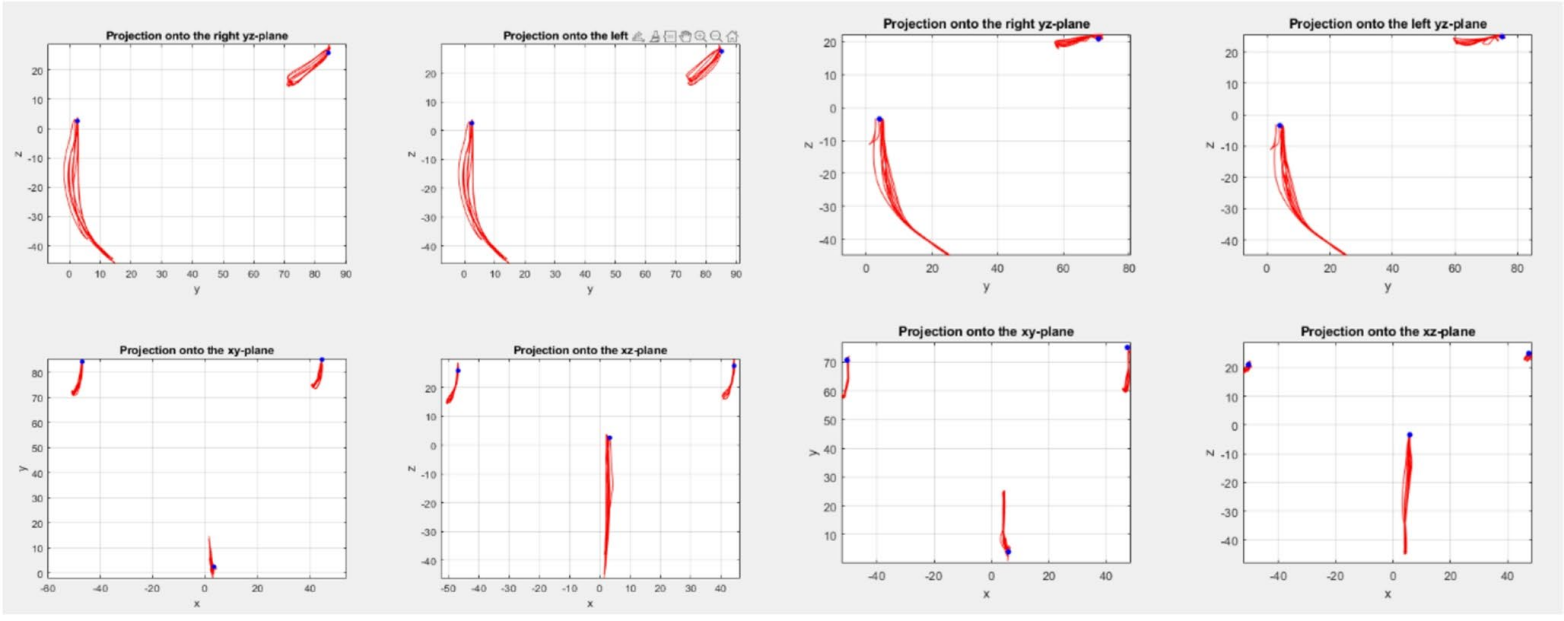
Exemplary samples of two cases showing the trajectories of the condyle centers and the incisal point in the respective planes and coordinate systems without the skull

The CoR was evaluated during mouth opening movements. The trajectories of the condylar center points and the incisal point was evaluated for all mouth opening movements for each participant and displayed in the occlusal plane (x-y-plane), the frontal plane (x-z-plane) and the right and left sagittal planes (y-z-plane) (Fig. 3). The start and end point of each condyle trace was marked and the difference vectors (∆y, ∆z) in the sagittal plane were calculated for both the right and left condyle. Additionally, the maximum opening angle ∆α was determined as the angle between a line between the condyle center point and the incisal point, when the mandible is in the habitual occlusion (line A), and a line between the condyle center point and the incisal point, when the mandible is in maximum opening position (line B). This opening angle ∆α was calculated for both the left and right condyle (*n* = 16). With these values (vector (∆y, ∆z) and angle ∆α) a theoretical CoR could approximately be estimated with an equation, previously derived and introduced (see Eq. (17) in Mehl (2020 Eq. (17)):

$$\begin{array}{cc}r_{y_0}=\frac{\mathrm\Delta y}2-\frac{\mathrm\Delta z}{\mathrm\Delta\alpha};&r_{z_0}=\frac{\mathrm\Delta z}2+\frac{\mathrm\Delta y}{\mathrm\Delta\alpha}\end{array};$$

with position $\:{r}_{{y}_{0}}$ and $\:{r}_{{z}_{0}}$ relative to the condyle center point [15].

This theoretically calculated CoR defined above was compared to the CoR determined by the Reuleaux method, as recommended in the literature [7, 17]. This was achieved using a previously established Matlab code [17]. The two reference points for this calculation were the incisal point and the frontal point of the chin (protuberancia mentalis), however, any other combinations of two points are also possible. The CoR was evaluated only for timeframes when the incisal point lied (1) within 3 mm from the incisal position in habitual occlusion, or (2) within 3 mm from the incisal position in maximum mouth opening. By extracting only these selected mandibular positions, the CoR represents the full (or nearly full) mouth opening movement. For each selected timeframe, the CoR was calculated from the paired positions of the two reference points with the Reuleaux method, resulting in between 50 and 100 measurements. These CoR positions were averaged, resulting in one CoR position for each participants maximum mouth opening movement (+/- 3 mm). This process was repeated for each participant.

A linear regression analysis was performed to evaluate the correlation between the calculated and measured CoR’s, from which the Pearson correlation coefficient was calculated (*p* < 0.05) (SPSS 30.0.0, IBM).

### Rotation-translation diagram

Mouth opening movements were further analyzed using rotation-translation diagrams. The position of the mandible was described by two parameters for each time frame including (i) translation of the condyle center, defined as the path length from the *start position* (rest position, habitual occlusion reference position) of the condyle center to the actual position of the condyle center and (ii) rotation of the mandible, defined as the angle between a first line (reference line) described by the *start position* of the condyle center and the incisal point and a second line defined by the *actual* position of the condyle center and the incisal point.

In contrast to a qualitive evaluation as proposed by Hugger et al. (2020) [1], where the traces were ranked visually according to 3 classes, linear regression lines were calculated for each condyle for (1) the entire opening movement and (2) three equally distributed sections (maximum angle/3). For each of these fitting lines the inverse inclination ∆s/∆α was calculated. For an easier interpretation, the values have been standardized to the unit mm per 10° degree. Values with a shift of less than 2 mm per 10° degree rotation, for example, indicate a motion which is predominantly guided by a rotation.

## Results

### Reference point trajectories

An example of traced trajectories for the condyle center and the 4 additional reference points are shown in Fig. 2. This representative example demonstrates the dependency of form and appearance of the trajectories from the selected reference points. With 3D segmented CBCT models, jaw motion tracking and custom visualization software, these effects could be better investigated in future and could serve as the basis for precision functional analysis, compared to conventional procedures using face bows and physical articulators or even electronic jaw tracking devices alone. With projections onto the respective planes, without displaying the anatomical structures, the trajectories can be visually analyzed further (Fig. 3), showing the traces of the condyle centers and incisal point.

### Centers of rotation

**Fig. 4:**
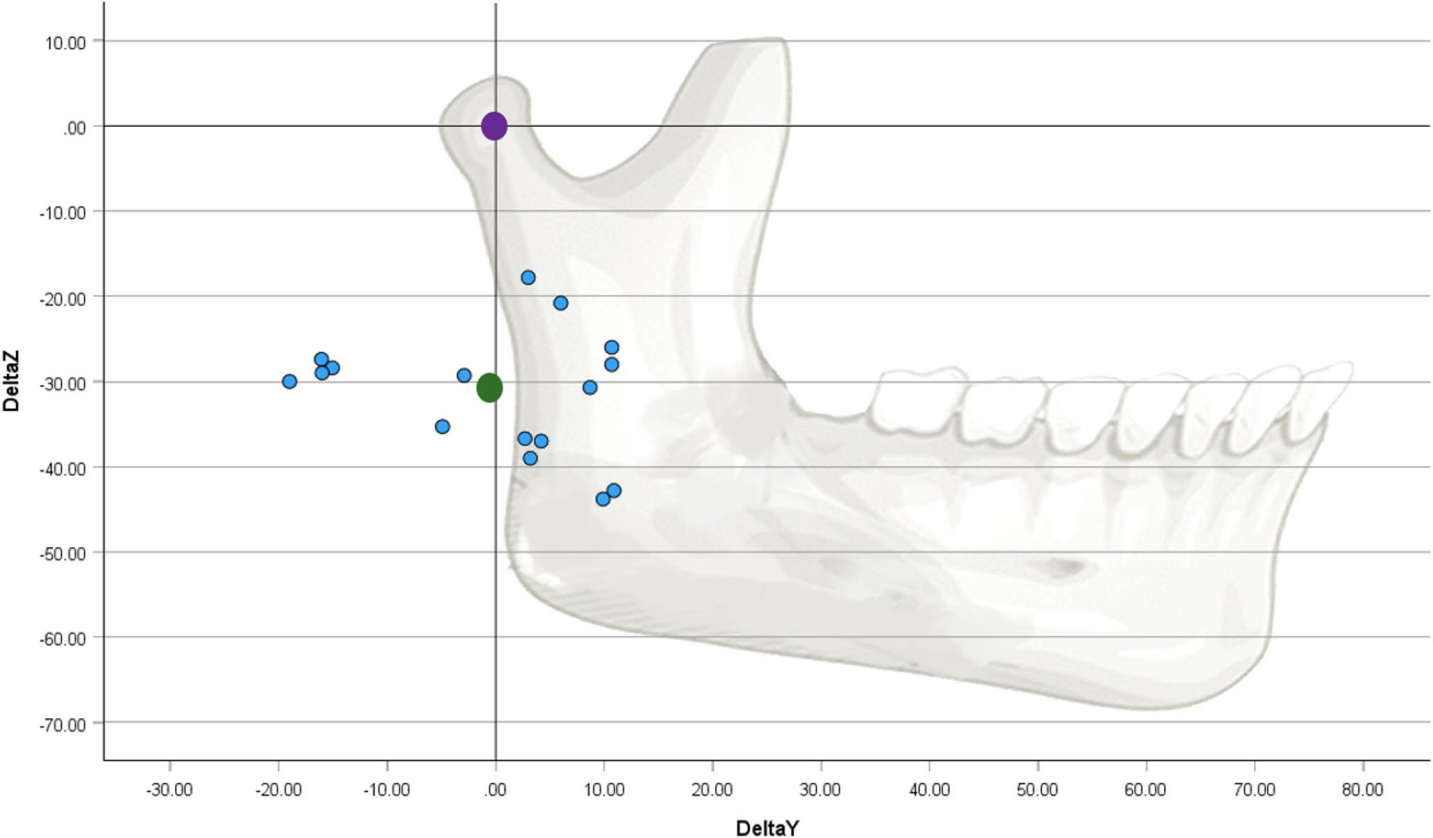
Distribution of the measured CoRs (centers of rotation, blue dots) relative to the condyle center (purple dots). The CoRs here were determined according to the Reuleaux method. The condyle position was chosen as the origin and a schematic mandible was drawn for better orientation. The average of the CoR locations are shown with the green dot (−32.4 mm, −0.2 mm)

The CoR of mouth opening movements, determined through the Reuleaux method with the clinically tracked data, were located on average caudally 32.4 (± 7.4) mm and 0.2 (± 10.8) mm posteriorly from the condyle center. The range was [19.8, 44.8] for the z-direction and [−19.0, 10.9] for the y-direction (Table 1). The distribution of all measured CoRs in relation to the fixed center of the condyle is schematically shown in Fig. 4. All CoRs are located around the occlusal plane (± 10 mm) and the posterior part of the ramus mandibulae (± 12 mm).

**Table 1:** Location of the center of rotation (CoR) in relation (∆y, ∆z) to the center of the condyle (CoC): Minimum, maximum, mean and standard deviation of the measured CoR (Measured values, Reuleaux method) and a theoretical approximation of the CoR calculated from the values of condyle displacement and opening angle (Calculated values, Theory)

Center of rotation (in reference to condyle center)	*N*	Minimum [mm]	Maximum [mm]	Mean [mm]	Std. Deviation [mm]
Measured ∆y (Releaux)	16	−19.0	10.9	−0.2	10.8
Measured ∆z (Releaux)	16	19.8	44.8	32.4	7.4
Calculated ∆y (Theory)	16	−19.8	16.7	−0.7	11.9
Calculated ∆z (Theory)	16	23.5	55.1	38.0	10.1

Theoretical CoRs, determined using only condylar traces during jaw opening, were positioned 38.0 (± 10.1) mm caudally and 0.7 (± 11.9) mm posteriorly from the center of the condyle. Similarly, the range was [23.5, 55.1] for the z-direction and [−19.8, 16.7] for the y-direction (Table 1). The calculated and the measured CoRs had comparable means and were lying within the same range (Table 1).

**Fig. 5:**
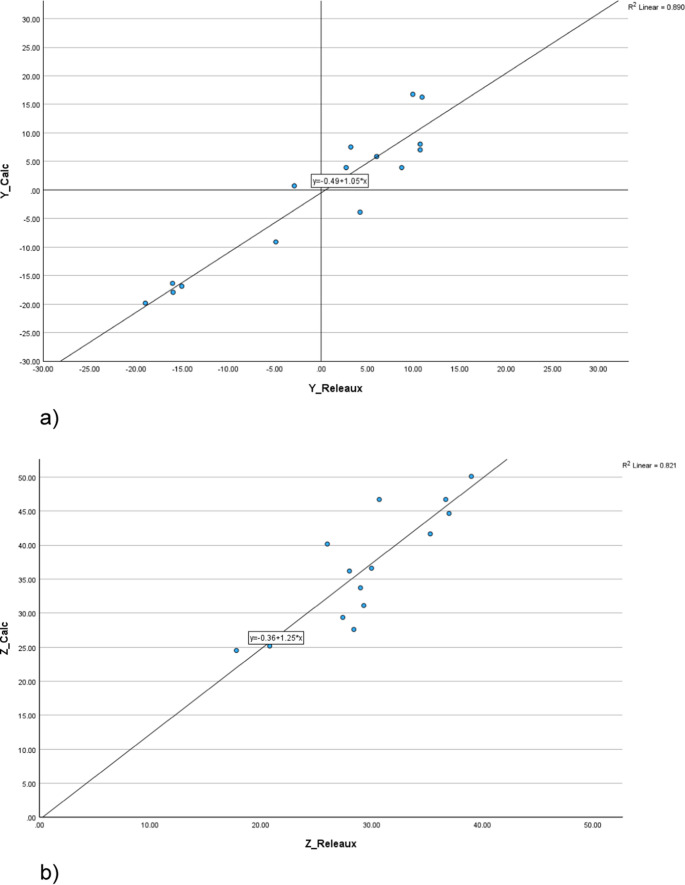
Scatter plot of the theoretically calculated versus Reuleaux determined y-values of the CoR (**a**) and the same for the z-values of the CoR (**b**). All values are relative to the center of condyle. In both diagrams a good linear correlation between the coordinates could be found (Pearson correlation coefficient *p* < 0.05)

A distinct correlation between the measured and theoretically calculated CoRs was determined by linear regression analysis (Fig. 5). The fitted linear function was 0.5 + 1.05*x with a correlation coefficient of R^2^ = 0.890 (*p* < 0.05), demonstrating a significant linear correlation. Similarly, the z-coordinates shows a linear relationship with R^2^ = 0.890 (*p* < 0.05) and a fitted linear function with 0.36 + 1.25*x. The theoretical equation was derived under the assumption of a linearly combined translation/rotation movement of the mandible during mouth opening. This strong correlation and the accurate prediction of the experimental data indicate that pure rotation during mouth opening is unlikely.

### Rotation-translation diagram

**Fig. 6:**
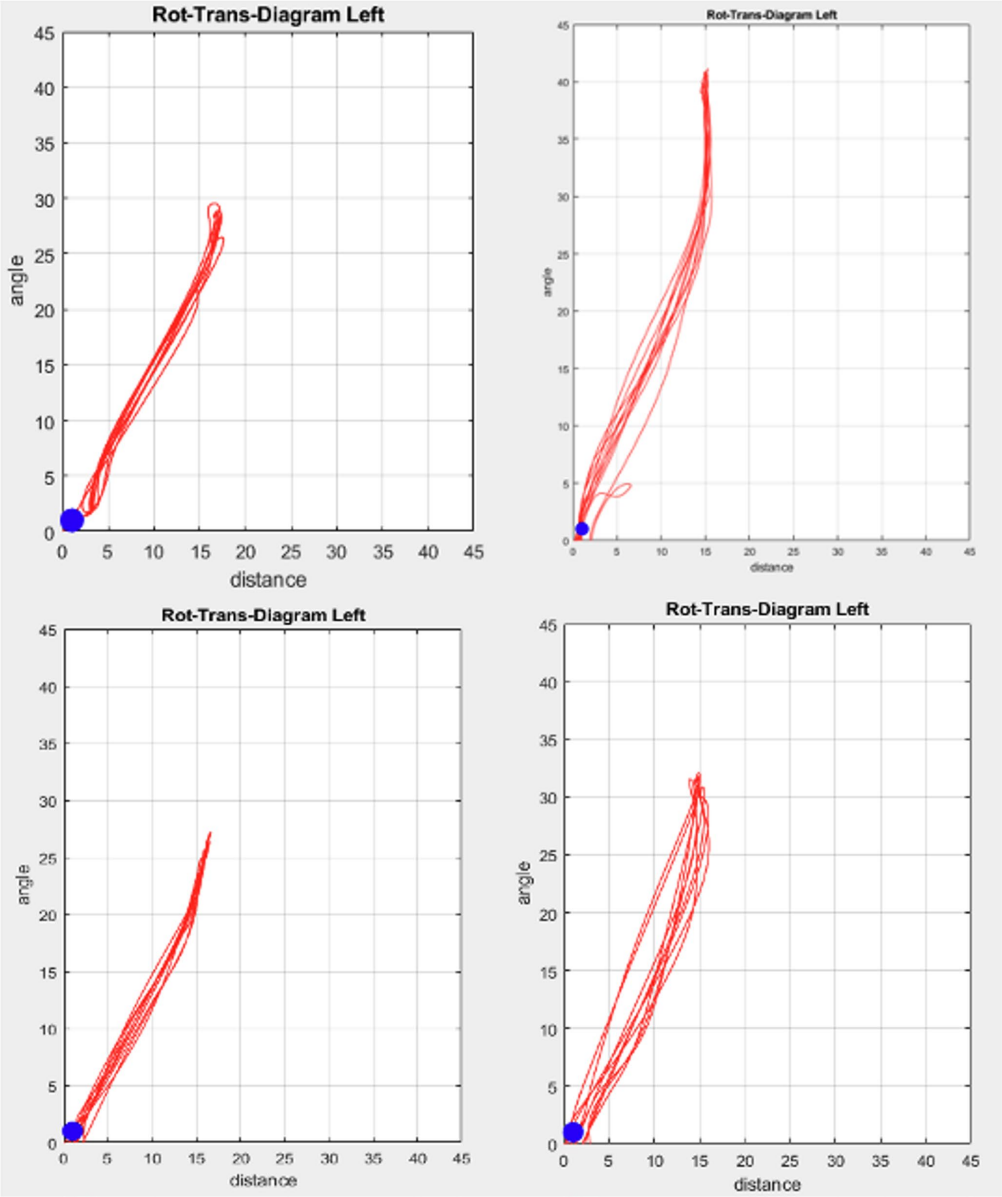
Four examples of a rotation-translation diagram: the two diagrams on the left side demonstrate a nearly linear behavior between the condyle shift (distance in mm) and condyle rotation (angle in ° degree). The upper right diagram shows an example with a starting linear behavior and a sigmoidal appearance at the end of the mouth opening, indicating a nearly pure rotation of the condyle. The lower right diagram represents a slightly irregular mouth opening-closing movement

Representative rotation-translation diagrams are shown in Fig. 6. Frequently observed curves exhibit linear behavior with a slight sigmoidal effect near the maximum opening position (Fig. 6). The inclinations for the linear fits are shown in Table 2. For the full mouth opening movement, the linear factor (inclination) averages to 5.51 (± 1.05) mm/10° degree. Therefore, on average there is a shift of the condyle of 5.5 mm for every 10 degrees of rotation. In one case, two movement segments could be detected, where the shift of the condyle is approximately 2 mm per 10° degrees of rotation (Table 2). These possible candidates for a pure rotation, however, are only present at the end of the mouth opening movement. When considering the initial 10 mm of opening (which in this study corresponds to an average opening angle of 7° (± 0.5°)), there were no cases where a translation of less than 3 mm was observed.

**Table 2:** Results of the linear regression of the rotation-translation diagrams

Translation/Rotation in mm/10°	Case 1	Case 2	Case 3	Case 4	Case 5	Case 6	Case 7	Case 8
Right condyle full	6.44	6.52	6.03	3.26	5.27	5.38	6.28	6.51
First part	6.89	9.46	7.13	3.48	7.27	5.43	9.51	7.28
Second part	6.96	7.89	7.19	4.42	5.05	7.55	4.76	7.11
Third part	7.06	8.38	4.37	2.01	3.17	3.39	8.79	5.45
Left condyle full	4.34	6.14	5.85	4.00	4.40	4.99	6.81	6.08
First part	6.30	10.45	7.76	5.49	6.70	4.54	9.88	6.07
Second part	5.00	7.49	6.27	5.16	4.21	6.43	6.25	7.18
Third part	5.24	6.95	6.66	0.98	4.94	4.91	6.40	6.51

For each condyle and each participant (case), linear fits were processed for the full mouth opening movement as well as for 3 equally distributed sections, the first (initial), the second and third part of the full mouth opening. The inclination values of the fitted lines are given in mm per 10°

**Fig. 7:**
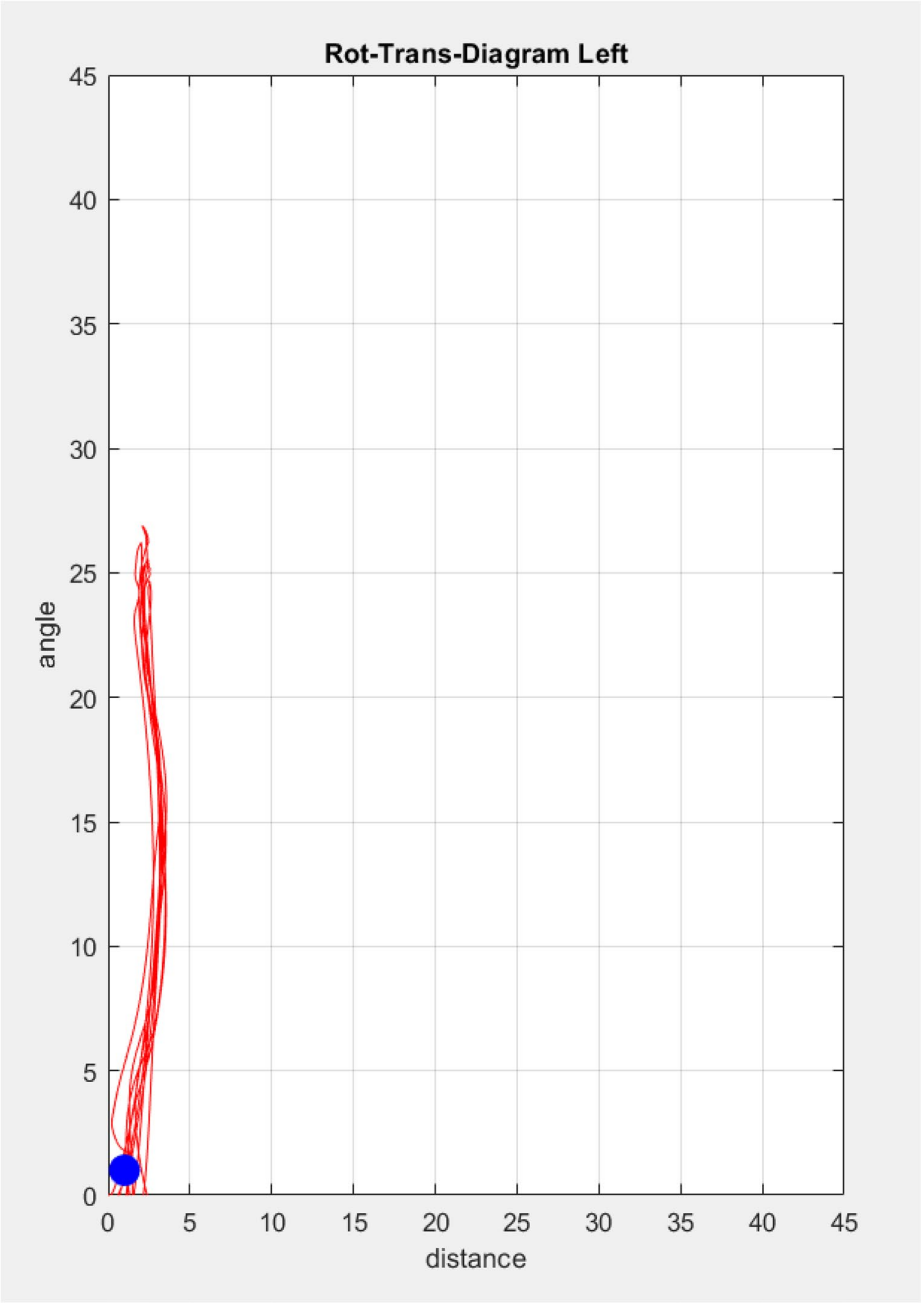
Rotation-Translation diagram when using the center of rotation (CoR) as the reference point instead of the condyle center. This indicates that the full mouth opening-closing movement can be approximately represented by a rotation around the CoR, with the translation of this point never exceeding 4 mm

In contrast, if the translation-rotation diagram is produced with the CoR as the reference point (instead of the condyle center), in many cases a good approximation of a pure rotation with only marginal translations can be observed (Fig. 7). These results provide strong evidence that during mouth opening, the condyle always exhibits a combined translational-rotational movement, and the (approximated) CoR is located far outside the range, where normally arbitrary condyle points are assumed.

## Discussion

This in vivo study developed a dynamic 3D virtual anatomical model, which allowed visualization and analysis of recorded mandibular movements. Anatomical reference points were interactively set which allowed 3D visualization of their movement trajectories. In this manner, the jaw movements of a set of healthy control participants were assessed by fusing optoelectronic motion data with subject-specific anatomical models segmented from CBCT scans. This software program can serve as the basis for future investigations of functional jaw movements.

The effect of cranial, caudal and dorsal offsets in condylar reference point positions, relative to the condyle center was evaluated. The appearance of the traces for mouth opening movements is clearly influenced by changes in cranial-caudal or anterior-posterior (ventral dorsal) directions in the sagittal plane and to a lesser extend in the frontal and horizontal planes. Whereas displacements in medial-lateral direction have little influence on the appearance of the trajectories. Although this is well-known behavior [2, 3, 16], the visualization in a virtual 3D model allows a clearer interpretation of such traces for both diagnostic and treatment purposes.

The CoR for the mouth opening movement was directly determined from the jaw motion measurements. To find a fixed CoR, the Reuleaux method was applied, which is a widely-used and accurate evaluation technique [7, 17]. A reliable calculation using the Reuleaux method is achieved if the distance between the two reference points attached to the mandible is sufficiently large [17]. In this study the position of the incisal point and the position of the chin (protuberancia mentalis) within one time frame were taken as the first point set. The same was done for a second point set after a defined opening angle. As the study aimed to find a fixed CoR for a nearly maximum mouth opening cycle, only data sets for the Reuleaux calculation were selected where the first point set corresponds to a mandible position near the incisal point (distance less than 3 mm) and the second point set corresponds to a mandible position near or at the maximum opening position (distance less than 3 mm). From all possible combinations of the two point sets, the CoR was calculated, and then all CoRs were averaged to determine a mean CoR. This approach reduces errors due to noise and accounts for the uncertainty of the exact maximum opening position.

The results from this study show that the CoR for a near maximum opening movement is located 32.4 ± 7.4 mm caudally (z-axis) and 0.2 ± 10.8 mm posteriorly (y-axis) from the condyle center. It is important to note that these values are related to a coordinate system where the xy-plane coincides with the occlusal plane, with the incisal point as the origin. A similar study found that the CoR lies, on average, caudally at 28 mm and posteriorly at 5.5 mm from the condyle center [7]. While these values slightly differ from those found in the current study, it must be noted that the results from Merema et al. (2022) [7] were calculated in a coordinate system oriented to the Frankfurt horizontal plane (FHP), which can be assumed to have an angle of around 10° to the occlusal plane [18]. A brief calculation for a coordinate transformation into the occlusal plane coordinate system results in values of 28.5 mm and − 0.5 mm, which are comparable to the results of this study. Interestingly, the CoR is located around the occlusal plane (average distance of around 32 mm to the condyle center [19]), and posterior to the ramus mandibulae, a finding that may warrant further investigation.

A theoretical calculation was made to interpret the results for the CoR. This theoretically derived formula assumed that the translation (∆s) of the condyle and the associated rotation of the mandible (∆α) are linearly correlated, meaning that the ratio of ∆s/∆α is constant for all time intervals [15]. Table 1; Fig. 5 show a strong correlation between the measured and theoretically calculated CoRs. Although this cannot be interpreted as a definitive proof, it strongly suggests that during the entire mouth opening movement, the rotation of the mandible is consistently associated with a translational component of the condyle movement. A pure rotation of the condyle (or around a CoR near the condyle axis) over a longer opening movement is unlikely.

Further evidence against a pure rotation during the initial opening movement can be extracted from the rotation-translation diagram. In all cases, for the first 10 mm of opening (corresponding to a rotation of the mandible with an angle of about 7°), the translational component of the condyle center is at least 3 mm. Even with this small translational component of 3 mm during the 10 mm mouth opening movement, the CoR (or in this case equivalent to an approximated instantaneous center of rotation) is located at least 20 mm from the condyle center [15], exceeding the assumed range of arbitrary condyle point locations. As all participants exhibited this behavior, the assumption that nearly all individuals experience a pure rotation of the mandible around an axis close to the condyle center can clearly be rejected. This makes the use of arbitrary face bows or functional concepts, which are relying on terminal hinge axis assumptions, questionable.

Moreover, the entire mouth opening movement shows an approximately linear relation between rotation angle and translational paths of the condyle. Although slight sigmoidal deviations at the end of the mouth opening movement were observed in a few cases, indicating that the rotational component increases with increasing mouth opening. Such observation are in good agreement with results from Hugger et al. 2020, who also showed nearly linear or slightly sigmoidal curves in the rotation-translation diagram in most of the investigated participants. However, the location of the reference points in their study could not be related to the condyles, possibly overestimating the amount of irregularly formed trajectories [1]. Therefore, based on the results of this study, it is worthwhile to conduct further research involving a larger sample size. This would support further understanding and confirmation of the biomechanical behavior of jaw movements.

## Conclusion

The following outcomes are associated with this study:

- A visualization software was established, which allows the fusion of segmented CBCT data and jaw motion tracking data. With that the jaw movements can be analyzed in every desired detail.
- The trajectories of landmarks show an explicit dependency from the spatial 3D-location in relation to the condyles. The functional analysis of arbitrary condyle point traces with conventional methods should be taken with caution.
- The center of rotation (CoR) for the mouth opening movements could be found to be located in average 32.4 (+/- 7.4) mm caudally and 0.2 (+/- 10.8) mm posteriorly from the condyle center (CoC). A theoretical calculation, assuming a linear combined rotation-translation movement of the condyles over the entire mouth opening results in a CoR 38.0 (+/- 10.1) mm caudally and 0.7 (+/- 11.9) mm posteriorly from the CoC. The correlation of measured and calculated CoRs over all participants was significant.
- The rotation-translation diagrams proof further the fact that for all mouth opening angles a rotation of the condyle is in general always associated with a translational movement of the condyle. In particular, for the initial mouth opening movement in no case a pure rotation of the condyle could be observed. The translational-rotational constant for the condyle movement can be approximated in average by 5.51 +/−1.05 mm per 10° degree.

## Data Availability

No datasets were generated or analysed during the current study.
